# Platelet-Derived Microvesicles Contribute to the Pathophysiogenesis of Human Cutaneous Leishmaniasis: A Nano-Flow Cytometric Approach in Plasma Samples from Patients before and under Antimonial Treatment

**DOI:** 10.3390/microorganisms12030526

**Published:** 2024-03-06

**Authors:** Vanessa Fernandes de Abreu Costa, Thaize Quiroga Chometon, Katherine Kelda Gomes de Castro, Melissa Silva Gonçalves Ponte, Maria Inês Fernandes Pimentel, Marcelo Rosandiski Lyra, Alvaro Luiz Bertho

**Affiliations:** 1 Laboratory of Immunoparasitology, Oswaldo Cruz Institute, FIOCRUZ, Rio de Janeiro 21040-360, RJ, Brazil; vanessafcsta@gmail.com (V.F.d.A.C.); thaizeqcp@gmail.com (T.Q.C.); melissaponte@live.com (M.S.G.P.); 2 Flow Cytometry Core Facility, Oswaldo Cruz Institute, FIOCRUZ, Rio de Janeiro 21040-360, RJ, Brazil; katherine.castro@ioc.fiocruz.br; 3 Laboratory of Clinical Research and Surveillance in Leishmaniasis, Evandro Chagas National Institute of Infectious Diseases, FIOCRUZ, Rio de Janeiro 21040-360, RJ, Brazil; maria.pimentel@ini.fiocruz.br (M.I.F.P.); marcelo.lyra@ini.fiocruz.br (M.R.L.)

**Keywords:** human cutaneous leishmaniasis, platelet-derived microvesicles, extracellular vesicles, flow cytometry, plasma, antimony treatment

## Abstract

Cutaneous leishmaniasis is a neglected tropical disease caused, in Brazil, mainly by *Leishmania braziliensis*, which is a protozoan transmitted during the blood feeding of infected female sandflies. To control leishmaniasis, the participation of CD4^+^ Th1 cells together with macrophages, neutrophils, and other peripheral blood cells, including platelets, is necessary. These anuclear fragments, when activated, produce microvesicles (MVs) that can reach locations outside the blood, carrying molecules responsible for activating pro-inflammatory responses and antigen presentation. Using flow cytometry, this current study evaluated the frequency and concentration of platelet-derived MVs (pMVs) in plasma samples obtained from patients in the acute phase and undergoing treatment, as well as from healthy volunteers. Our results revealed a higher frequency and concentration of pMVs in the plasma of patients with acute CL when compared to all other groups studied. These results highlight the impact of pMVs in modulating the immune response of CL patients, correlating their higher concentrations and frequencies in CL-patient plasmas, with the acute inflammatory status of the disease and their reduction with beneficial results of systemic treatment with antimony. This knowledge is essential to define potential treatment protocols, as well as highlight pMVs as biomarkers for the different clinical stages of CL.

## 1. Introduction

Tegumentary leishmaniasis (TL) is among the world’s top ten neglected tropical diseases and one of the most important for public health. TL is endemic in 89 countries, with more than 12 million people infected and 600,000 to 1 million new cases annually [1]. In the region of the Americas, cases have been reported from the south of the United States to the north of Argentina, except for the Caribbean islands and Chile, with 19 countries being endemic for TL [1,2]. In Brazil, TL is caused mainly by *Leishmania (Viannia) braziliensis* and is transmitted through the blood meal of infected sandflies of the genus *Lutzomyia*. Around twenty thousand people presented TL annually, with a large part of the registrations occurring in rural areas, where structural investments in sanitation, health care, and basic education are less frequent [3,4]. The disease presents a wide spectrum of clinical forms of varying severity, which is determined by the type and magnitude of the host immune response, the genetic background of the individual, and the parasite species responsible for the infection. According to its clinical status, TL is classified as cutaneous leishmaniasis (CL), with localized cutaneous lesions, and mucosal leishmaniasis (ML), presenting occasionally severe mucosal deformations. Lesions may begin as papules or nodules during acute infections, showing an important inflammatory pattern, and persist as chronic infection through a varied period of time, which, once cured under treatment or spontaneously, may leave disfiguring scars [1,2]. 

During the blood-feeding activity by infected female sandflies, promastigotes forms of *Leishmania* spp. are introduced in the epidermis, stimulating an innate immune response, with an influx of leukocytes, mainly macrophages and neutrophils, and platelets, causing a strong inflammatory process. This process leads to the release of antigenic particles that will be presented to the immune system, generating the specific acquired immune response, orchestrated mostly by T lymphocytes. It is probably at this moment that characteristics such as intensity and quality of the immune response are defined, thus influencing the progression of the disease towards a self-limited and spontaneous cure or progressive forms [5]. It is already well known that the predominance of the cellular immune response necessary to resolve the infection consists of CD4^+^Th1 lymphocytes producing cytokines such as IL-2, IFN-γ, and IL-12. This response results in the activation of macrophages, making them capable of eliminating the parasite and controlling the infection. On the contrary, anti-inflammatory cytokines such as IL-4, IL-10, and TGF-β released by CD4^+^ Th2 cells inhibit leishmanicidal immune response, leading to the persistence of parasites and chronicity of lesions [6,7]. It is not fully understood how CD8^+^ T cells are activated in leishmaniasis, but it is clear that they respond during *Leishmania* infection, acting in a bi-modal manner, either beneficiating host immune response [8,9,10,11,12,13,14] or worsening the progression of the disease [8,15,16].

The most used treatment in the Americas comprises topical and systemic treatment with pentavalent antimony compounds of meglumine antimoniate and sodium stibogluconate (Sb^5+^) drugs. In Brazil, localized and disseminated cutaneous forms can be treated with meglumine antimoniate 10 to 20 mg of Sb^5+^/Kg/day, with 15 mg of Sb^5+^/Kg/day suggested for both adults and children during 20 consecutive days. If there is no complete healing within three months after the end of treatment, the scheme must be repeated, extending the duration of the treatment to 30 days. Alternatively, in Rio de Janeiro, patients can be initially treated with meglumine antimoniate 5 mg Sb^5+^/Kg/day for 30 days. Intralesional meglumine antimoniate is sometimes used, especially when the patients have heart, kidney, or liver disease [1,17,18,19]. Some combined therapies have been highlighted to be used for the treatment of CL lesions, such as lesion-inoculation of platelet-rich plasma, as well as the topical use of autologous platelet gel [20,21].

Platelets, also called thrombocytes, are anucleated, discoid-shaped cytoplasmic structures present in the blood that act to prevent hemorrhages through the clotting process. They have significant participation in modulating hemostasis, thrombosis, inflammatory reactions, and immune response by carrying and delivering pro-inflammatory cytokines (e.g., IL-1 and IL-10) and growth factors (platelet-derived growth factor—PDGF), which are chemotactic for neutrophils, monocytes, and lymphocytes, attracting them to the site of inflammatory lesions [22]. Moreover, some studies have shown that, under activation, platelets produce and release extracellular vesicles (EVs), which act as their precursor cells in homeostasis and coagulation. EVs are heterogeneous structures surrounded by phospholipid membranes, being classified based on their relative dimensions, biogenesis, and cargo contents. Thus, they are defined as microvesicles (MVs), which are generated by the budding of the plasma membrane, ranging from 100 to 900 nm and presenting phosphatidylserine (PS) exposure (Annexin-V^+^ binding); exosomes, the smallest EVs (40–100 nm), which are derived from exocytosis of multivesicular bodies and granules; and apoptotic bodies (>1000 nm) released like blebs from apoptotic cells [23,24,25]. The formation and release of EVs seem to relate to cellular homeostasis by balancing intra- and extracellular signals. Noticeably, EVs are expected to contribute to physiology and pathology, serving as potential clinical biomarkers for the diagnosis, prognosis, and therapy of certain diseases [23,24]. 

Platelet-derived EVs (pEVs) have important roles in hemostasis, coagulation, immune response, and inflammatory processes. pEV cargo is incredibly diverse and can include lipids, proteins, nucleic acids, and organelles involved in numerous other biological processes. Furthermore, while platelets cannot cross tissue barriers, pEVs can enter lymph, bone marrow, and synovial fluid, allowing the transfer of platelet-derived content to cellular recipients and organs inaccessible to platelets, contributing to the dissemination of platelet components into tissues and organs localized in sites beyond the blood system [26,27]. They also participate in the inflammatory process through their impact on cell–cell interaction, carrying pro-inflammatory cytokines, such as IL-1, IL-6, and TNF-α, and their contribution in inducing the expression of adhesion molecules and the release of cytokines by different cell types [27]. It was already stated that over-activated platelets and increased pEVs circulation in the blood were associated with high inflammatory responses, thrombosis, and multiple organ damage in COVID-19 patients [28]. Another investigation has also suggested that pEVs could infiltrate the bone marrow during an inflammatory state and promote hematopoiesis [29]. A recent study indicated that tissue cells and macrophages interact via the release of small EVs, promoting the activation and polarization of adjacent macrophages, which can in turn release EVs and factors that can promote cell stress and tissue inflammation and injury [30]. In the lymph, they circulate in the absence of inflammation, suggesting that this fluid, absent of any platelets, is used by pEVs to reach tissue locations inaccessible to the platelets themselves [26]. 

To date, there are no systematic studies on the roles and corresponding mechanisms of pEVs in the pathophysiology of CL, and most of the articles found on this issue focused on investigating the impact of the protozoan-derived EVs in the host immune responses [31,32,33]. In order to address this gap in knowledge, we investigated the contribution of pMVs in the pathophysiology of acute cutaneous leishmaniasis and during antimony treatment. For this, we investigated the concentrations and frequencies of pMVs in plasma samples from patients of acute CL and from those submitted to pentavalent antimonial systemic treatment, using nano-flow cytometry. 

## 2. Materials and Methods

### 2.1. Patients

This study included 40 patients with localized cutaneous leishmaniasis (CL), as confirmed by parasite detection (culture, PCR), which were all residents in a CL endemic area of the state of Rio de Janeiro, Brazil, and they were divided into 3 cohorts: PBT—18 patients before meglumine antimoniate treatment, characterizing the group with acute disease; PDT—12 patients on day 10/13 during treatment; and PPT—10 patients between day 70 and 95 after the beginning of treatment, patients considered clinically cured, according to criteria defined by the medical staff of the Laboratory of Clinical Research and Surveillance in Leishmaniasis, Evandro Chagas National Institute of Infectious Diseases, Oswaldo Cruz Foundation (LVL/INI/FIOCRUZ). The study also included HI—10 healthy individuals without a previous diagnosis of leishmaniasis and not living in endemic areas for CL. The diagnosis of the disease and the outpatient follow-up of the patients were carried out at the outpatient clinics of LVL/INI/FIOCRZ, Rio de Janeiro, Brazil. All patients had the diagnosis of cutaneous leishmaniasis confirmed by one of more of the following techniques: culture in NNN plus Schneider’s drosophila media and direct smear examination through optical microscopy (Giemsa stain) (1); immunohistochemistry [34]; and kDNA PCR, using primers specific for *L. braziliensis* [35]. The CL-specific treatment consisted of the use of meglumine antimoniate administered intramuscularly at 5 mg Sb^5+^/Kg/day for 30 days, as previously described [1,17,18,19]. The exclusion criteria adopted were as follows: age under 18 and over 60 years; presence of comorbidity (e.g., HIV, diabetes mellitus, neoplasia, autoimmune diseases, or hepatitis); pregnancy; and previous treatment with anti-*Leishmania* drugs. Differential diagnosis of other cutaneous diseases through mycological and bacteriological tests was also performed. Furthermore, none of the patients showed progression to mucosal or disseminated clinical forms. All patients at the end of treatment (PPT) presented clinical cure, which was characterized by full epithelialization of ulcerated lesions, and regression of crusts, desquamation, and infiltration.

This study was approved by the Health Ethics Committee of the Oswaldo Cruz Institute (Plataforma Brasil), CEP FIOCRUZ/IOC, no. 2.917.449 of 28 September 2018. All individuals who voluntarily agreed to participate in this project signed, before blood collection, the Free and Informed Consent Form, written in accordance with the norms of Resolution 466/2012 of the National Health Council, Ministry of Health, Brazil. Patients who did not sign the Informed Consent Form were excluded from the study.

### 2.2. Reagents

Annexin V-FITC (BD Biosciences, Franklin Lakes, NJ, USA) and calcein AM violet (Biolegend, San Diego, CA, USA) were used to define MVs; CD41-PE and CD235a monoclonal antibodies (Beckman Coulter, Brea, CA, USA) for MVs phenotyping; IgG1 Mouse-PE isotype control (Beckman Coulter); annexin V binding buffer (BD Bioscience); Megamix-Plus FSC (100, 300, 500, and 900 nm); and Megamix-Plus SSC (160, 200, 240, and 500 nm) beads (BioCytex, Marseille, France) were used to define relative size of MVs.

### 2.3. Blood Collection and Plasma Preparation

To date, there are some standardized flow cytometry protocols for analyzing EVs in human plasma samples, but they still present some controversial points. Some issues, mainly in pre-analytical procedures (e.g., blood collection and delay time between collection and sample processing/analysis), need to be standardized and adapted to daily clinical routines in hospitals to streamline plasma processing procedures and the start of analysis by flow cytometry. Thus, we adapted and standardized the pre-analytical and sample processing procedures based on those described elsewhere [36] and reported according to the criteria established by the MISEV guidelines [37,38]. Briefly, blood samples were collected in 5 mL 0.109 M citrated plastic tubes (BD Vacutainer, Becton Dickinson) via antecubital vein puncture using a 21-gauge needle, without the use of a tourniquet and without agitation. The first 1 mL was discarded prior to collection of 3.5 mL of blood. Tubes were carefully transported vertically at room temperature, without agitation. Within 1 h of blood withdrawal, platelet-depleted plasma was prepared as follows: centrifugation at 300× *g* for 10 min at 20 °C, with acceleration and brake to obtain the plasma (supernatant); centrifugation of the supernatant at 2500× *g* for 15 min at 20 °C, with the lowest deceleration, to obtain platelet-poor plasma (PPP). Subsequently, the PPP was gently transferred into a polypropylene tube and centrifuged at 13,000× *g* for 5 min at 20 °C, with acceleration and brake, to obtain platelet-free plasma (PFP). The PFP was distributed into 50 μL aliquots and submitted immediately to the flow cytometry labeling protocol.

### 2.4. Nano-Flow Cytometry Analysis of Extracellular Microvesicles

The nano-flow cytometry was performed using a CytoFLEX flow cytometer (Beckman Coulter) equipped with 405 nm, 488 nm, and 638 nm lasers to detect up to 12 fluorescence parameters. Extracellular vesicles and other biological nanoparticles (NPs) generally fall within the optical noise of conventional flow cytometry, and most flow cytometers are not sensitive enough to effectively detect NPs less than 300 nm in diameter. The nano-flow cytometry is sensitive to this purpose because the SSC detectors actually have an attenuation filter to reduce 95% of the scatter signal and Violet SSC (VSSC) can be used to bring the sensitivity well into the nanoparticle range. Shorter wavelengths, such as 405 nm (violet), result in greater orthogonal particle light scattering, as well as increase their resolution range. In this way, particles such as EVs, that are nanometric in size, have their refractive index amplified using the violet laser, thus facilitating their detection [39]. To adjust the CytoFlex for MV measurements, the standard filter configuration was changed so that the VSSC was used as a trigger signal to discriminate the noise instead of the normally used 488 nm FSC. The changes in filter configuration to detect MVs were as follows: in front of 405 nm-laser APD sensors, the 450/45 nm-filter position was changed to 405/10 nm one; the 525/40 nm filter was changed to a 450/45 nm filter position; and the 610/20 nm filter was changed to a 525/40 nm filter position. All filters from 488 nm to 638 nm lasers remained as default-conventional CytoFlex setup. To reduce the background noise, this flow cytometer permits dual threshold settings in the Violet SSC and the FITC channels or Blue SSC. The VSSC threshold was set as the trigger channel below the 100 nm bead population, which gave us an acceptable noise of about 30–200 events/s. For daily routine, the flow line was cleaned with 0.2 µm filtered Coulter Clenz (Beckman Coulter) for 5 min, followed by type 1 ultrapure water (Merck, Darmstadt, Germany) for 15 min. The type 1 ultrapure water (Merck) was used as sheath fluid, and to avoid carry-over effects between each sample measurement we performed a washing step with 0.2 µm filtered type 1 ultrapure water for 2 min at an increased flow rate of 60 mL/min. 

The equivalent nanometric sizes of MVs were established by acquiring a mix of size-defined polystyrene beads, Megamix-Plus FSC (100, 300, 500, and 900 nm), and Megamix-Plus SSC (160, 200, 240, and 500 nm) (BioCytex, Marseille, France) through a nano-flow cytometric protocol comprising a Blue SSC-logH vs. Violet SSC-logH dot plot, where a rectangular gate (P1) was set, encompassing the bead populations (Supplementary Figure S1A), and a Violet SSC-logH histogram gated on P1, which showed 8-Gigamix-bead peaks with diameters ranging from 100 to 900 nm (Supplementary Figure S1B). 

For MVs nano-flow cytometry analysis, an aliquot of PFP (10 μL) was labeled with a mix of reagents containing the following: 2.5 µL of annexin V-FITC, for phosphatidylserine binding (BD Bioscience); 2.5 µL of calcein AM violet (Biolegend), for particle membrane integrity; 3 µL of anti-CD41a-PE monoclonal antibody (mAb) (Clone: P2; Beckman Coulter, Indianapolis, IN, USA), for platelet-derived MV (pMV) staining; and 3 µL of anti-CD235a APC Fire 750 mAb (Clone HIR2; Biolegend), for erythrocyte-derived MV (eMV) staining, which was diluted in a final volume of 50 µL of 0.2 μm filtered annexin V binding buffer (Anx-bB–BD, Bioscience) (mix-sample). After the 30 min incubation at 20 °C, the mix-sample was diluted to the final volume of 2 mL of Anx-bB to avoid swarm effects, and it was acquired immediately in the flow cytometer. Prior to the staining, the antibodies/annexin V/calcein mixture was centrifuged at 20,000× *g* for 30 min at 4 °C to remove fluorescent aggregated particles [40,41]. All samples were acquired at low speed, where the level of events did not exceed 300 events/s and the acquisition flow rate was 10 µL/min for a period of 1 h or the maximum of events was reached within the gate. 

All intra-assay quality controls were used in accordance with regulations described elsewhere [37,38]: (1) 0.2 µm-filtered Anx-bB-only control; (2) Anx-bB + mix (antibodies, annexin, and calcein)-reagents control: to ensure the removal of fluorescent background particles; (3) isotype control: 3 µL of PE isotype (Beckman Coulter) pre-centrifuged at 20,000× *g* for 30 min was used; (4) detergent lyse control: 10 µL of NP-40 (IGEPAL CA-63, Sigma-Aldrich, St. Louis, MO, USA) was added to a mix-sample tube, after acquisition and reacquiring after 1–2 min, to validate the true-MVs. Flow cytometer acquisition settings were maintained for all samples, including triggering threshold, gains, and flow rate. 

### 2.5. Data Analysis and Statistics

Flow cytometric analyses were performed using the software CytExpert v. 2.3 (Beckman Coulter), where we applied gates of interest to evaluate the percentage/frequency of MVs, as well as the number of events per µL to evaluate MV concentration. Statistical analysis was performed with GraphPad Prism 7.0 software (GraphPad, San Diego, CA, USA). The Mann–Whitney U-test was used to compare the two groups for unpaired observations. Probability levels of *p* values ≤ 0.05 were considered statistically significant. 

## 3. Results

### 3.1. Characteristics of CL-Patient Cohorts and Healthy Individuals

Table 1 summarizes some clinical and epidemiological data of the patients enrolled in this study. The majority of CL patients were male (57.5%), with a mean age ± standard deviation (SD) of 36.08 ± 15.04 years. The number of lesions ranged from one to five, with a diameter ranging from 14 to 130 mm. The mean age ± SD of this group was 29.6 ± 10 years, and we had 50% of individuals for each sex (Table 1).

### 3.2. Standardization of the Blood Collection Procedures for Improvement of Analysis of MVs in the Human Plasma

To evaluate the impact of the anticoagulants in the improvement of concentration of MVs in human PFP samples, we obtained FPF from blood collected with anticoagulants—heparin, EDTA, and sodium citrate—and analyzed through nano-flow cytometry. We observed that the plasma sample obtained from the tube containing the sodium citrate presented a higher concentration of MVs (5.19%) within the calcein/annexin V-double positive (True-MVs) gate.

Still, we investigated whether the freezing process could impact MV concentration in plasma samples stored at −80 °C for 1 day or at least 60 days. We observed low levels of MVs in plasma samples defrosted from both 1 day at −80 °C and >60 days at −80 °C storage protocols when compared to fresh plasma samples. This observation suggested that the freezing procedure reduced the formation of aleatory MVs, and to avoid this, we performed all the experiments with fresh plasma samples (Supplementary Figure S2).

### 3.3. Definition of a Cytofluorimetric Gate Strategy Protocol for Quantification and Phenotyping of MVs in Platelet-Free Plasma Samples from CL Patients and Healthy Individuals

**Figure 1:**
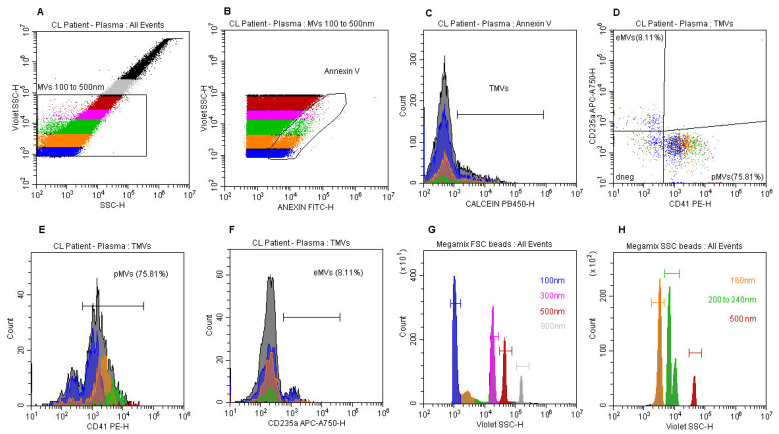
Flow cytometry representative protocol for detection and phenotyping of extracellular microvesicles from the plasma of the studied groups. A gating strategy was performed as follows: (**A**) dot plot of Blue SSC-log-H vs. Violet SSC-log-H, where a gate (MVs) englobing 100–500 nm size MVs was created; (**B**) dot plot of Annexin-FITC-log-H vs. Violet SSC-log-H, gated on MVs 100–500 nm, where a gate (annexin V) encompassing annexin V^+^-MVs was created; (**C**) histogram of calcein AM-PB450-log-H gated on annexin V to determine true MVs (TMV gate); (**D**) dot plot of CD41-PE-log-H vs. CD235a-APC/AF750-log-H to determine platelet-derived MVs (pMVs) and erythrocyte-derived MVs (eMVs), respectively; (**E**) histogram of CD41-PE-log-H to determine pMVs; (**F**) histogram of CD235a-APC/AF750-log-H to determine eMVs; (**G**,**H**) histograms of Violet SSC-log-H defining the relative sizes of Megam9ix FSC and SSC beads, respectively. Flow cytometric analytic template created using CytoExpert software v. 2.3 (Beckman Coulter).

For assessment of frequencies (%), concentrations (events/µL) and phenotyping of the MVs in plasma samples from CL patients and healthy individuals, the followinga flow cytometry gate strategy protocol was designed, (Figure 1): dot plot Blue SSC-log-H vs. Violet SSC-log-H, where was created a gate encompassing 100–500 nm-size MVs (Figure 1A), based on relative sizes obtained from Megamix FSC and SSC beads (Figure 1G and Figure 1H respectively); dot plot annexin-FITC-log-H vs. Violet SSC-log-H, gated on MVs 100–500 nm, where was created a gate (annexin V) encompassing annexin V^+^-MVs (Figure 1B); histogram calcein AM-PB450-log-H gated on annexin V to determine true MVs (TMVs gate) (Figure 1C); dot plot CD41-PE-log-H vs. CD235a-APC/AF750-log-H to determine frequencies (%) of platelet-derived MVs (pMVs) and erythrocyte-derived MVs (eMVs) (Figure 1D); histogram CD41-PE-log-H to determine the frequencies (%) of pMVs (Figure 1E); histogram CD235a-APC/AF750-log-H to assess the frequencies of eMVs (Figure 1F); histograms Violet SSC-log-H defining the relative sizes of Megamix FSC and SSC beads (Figure 1G and Figure 1H, respectively).

The evaluation of MV concentrations (events/µL) was carried out using the same pro-tocol, changing percentage to events/µL.

### 3.4. Evaluation of the Frequencies and Concentration of MVs in Plasma Samples from CL Patients and Healthy Individuals

The frequency (%) and concentration (events/µL) of MVs in the plasma samples from healthy individuals and from patients at different times of treatment, PBT (patients before treatment), PDT (patients during treatment), and PPT (patients at the end of treatment), were analyzed through nano-flow cytometry.

**Figure 2:**
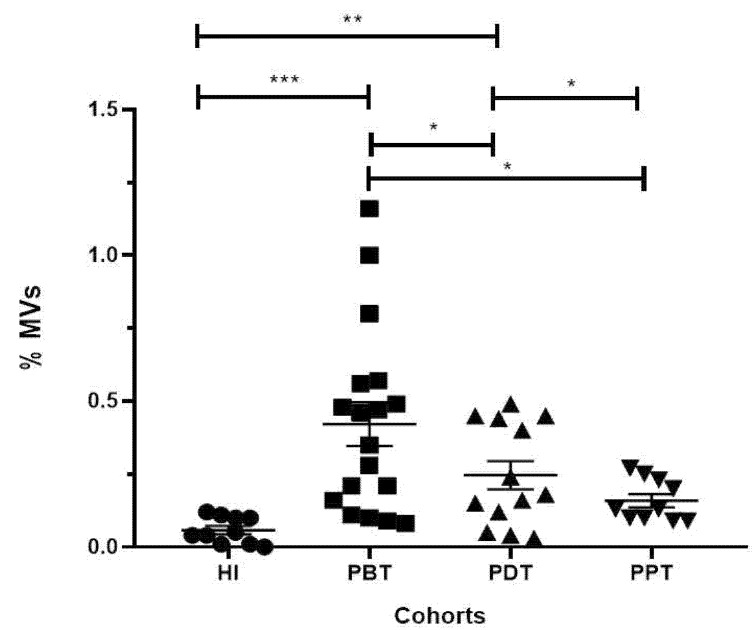
Scatterplots of the percentage of MVs in PFP samples from healthy individuals and CL patients. HI (healthy individuals, *n* = 10) and CL patients in different phases of treatment: PBT (patients before treatment, *n* = 18), PDT (patients during treatment, *n* = 12), and PPT (patients at the end of treatment, *n* = 10) were evaluated. Data are represented by mean and standard error. Each dot represents an individual. Statistical comparisons were performed using the non-parametric Mann–Whitney U-test, where * indicates *p* ≤ 0.05; ** indicates *p* ≤ 0.01; *** indicates *p* ≤ 0.001.

When we evaluated the frequencies of MVs (%) in the groups studied, we observed higher frequencies (%) in the PBT group (mean ± SEM = 0.42 ± 0.07; *n* = 18) compared to the HI group (0.06 ± 0.01; *p* < 0.0001; *n* = 10), PDT group (0.25 ± 0.05; *p* = 0.0398; *n* = 13), and PPT group (0.16 ± 0.02; *p* = 0.0199; *n* = 10) (Figure 2). These data strongly suggest that acute CL should be related to high MV frequencies, and, when patients were submitted to antimony therapy, there was a decrease in MV frequencies, leading to physiological levels at the end of treatment.

**Figure 3:**
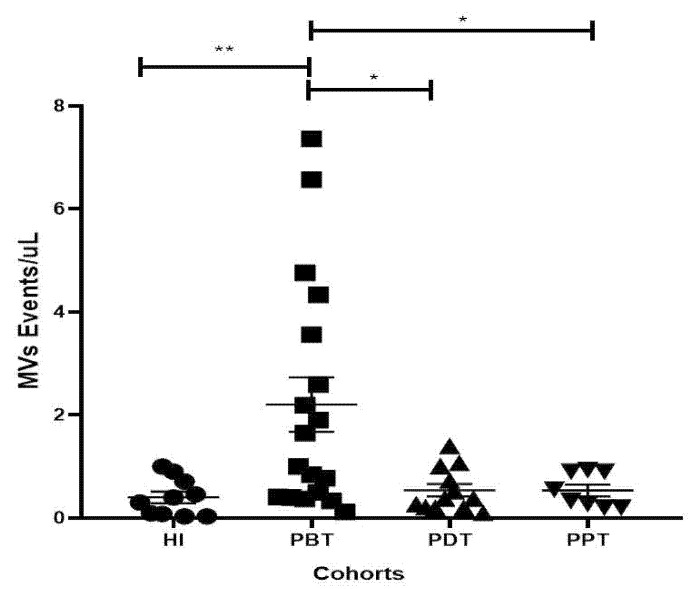
Scatterplots of the concentration (events/μL) of MVs in PFP samples from healthy individuals and CL patients. HI (healthy individuals, *n* = 10) and CL patients in different phases of treatment, PBT (patients before treatment, *n* = 18), PDT (patients during treatment, *n* = 12), and PPT (patients at the end of treatment, *n* = 8), were evaluated. Data are represented by mean and standard error. Each dot represents an individual. Statistical comparisons were performed using the non-parametric Mann–Whitney U-test, where * indicates *p* ≤ 0.05; ** indicates *p* ≤ 0.01.

In another type of approach that evaluates the concentration (events/μL) of MVs in the plasma sample of the studied groups, we can observe that there is a higher MV concentration in the PBT group (mean ± SEM = 2.20 ± 0.52; *n* = 18) compared to the HI group (0.40 ± 0.12; *p* < 0.0042; *n* = 10), PDT group (0.54 ± 0.121; *p* = 0.011; *n* = 12), and PPT group (0.54 ± 0.12; *p* = 0.0364; *n* = 8) (Figure 3). These data corroborate those stated above, suggesting that higher MV concentrations are associated with an inflammatory profile found in the acute CL, which reverted to a low MV concentration when patients were submitted to therapeutic protocols. 

### 3.5. Phenotyping of MVs in Plasma Samples from CL Patients and Healthy Individuals

**Figure 4:**
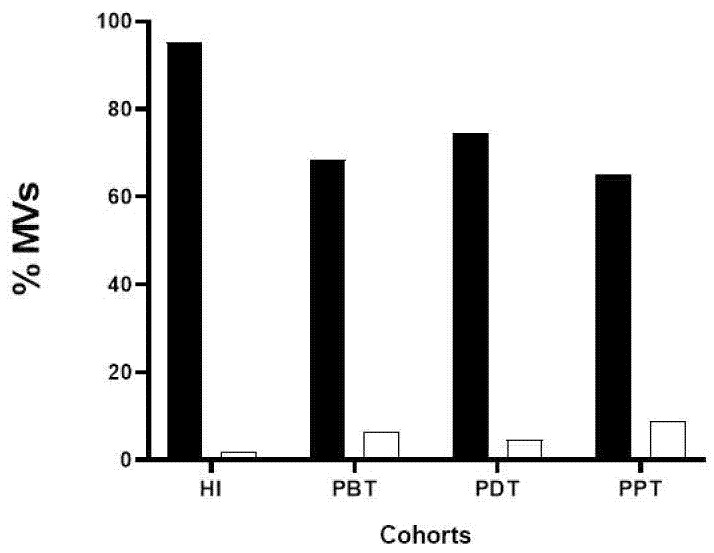
Phenotyping to define the cell origin of MVs in the plasma of healthy individuals and patients in different phases of treatment. Bar graphs showing the median percentage of platelet-derived MVs (solid bar—CD41^+^MVs) and erythrocyte-derived MVs (empty bars—CD235a^+^ MVs) in plasma samples from HI—healthy individuals; PBT—patients before treatment; PDT—patients during treatment; and PPT—patients at the end of treatment.

We also analyzed the cell origin of MVs found in the plasma samples from CL patients and healthy individuals and their respective sizes. We labeled MVs with anti-CD41 monoclonal antibodies to define platelet-origin MVs and anti-CD235a moAb to erythrocyte-origin MVs. It was observed that the majority of MVs present in plasma were predominantly derived from platelets (75.78 ± 13.48%) in all groups studied (Figure 4). 

## 4. Discussion

Cutaneous leishmaniasis (CL) in Brazil is mainly caused by *L. (Viannia) braziliensis,* which is a phagosomal protozoan transmitted to humans during blood meals by infected sandflies, infecting dermal resident macrophages, neutrophils, and dendritic cells, leading to the formation of inflammatory lesions on the skin at the site of infection [1,2]. Clinical manifestations occur due to the immune response triggered by the host, which can contribute to the cure or exacerbation of the disease [5]. Local and systemic treatment with pentavalent antimony compounds of meglumine antimoniate (Sb+5) drugs is the protocol suggested by the Brazilian Ministry of Health [1,17,18,19]. Platelets are important in the pro-inflammatory process to resolve CL lesions. Therefore, some combined therapies have been used for the treatment of CL lesions, such as lesion-inoculation of platelet-rich plasma [20,21]. For this process to be effective and successful, the immune response cells must be able to communicate efficiently with each other. Such communication occurs through mechanisms like the production and release of cytokines, chemokines, growth factors, and extracellular vesicles (EVs). EVs are membrane vesicles released from the cellular plasma membrane (microvesicles—MVs), endosomal compartment (exosomes), or after cell deaths (apoptotic bodies). The isolation and characterization of EVs, which are formed and secreted by normal and pathological cells, has become a promising method to improve the understanding of the pathophysiology of some diseases and inflammatory processes [23,24,25]. Platelets upon activation produce EVs that have been associated with both noninfectious chronic inflammatory diseases (e.g., sepsis, atherosclerosis, diabetes mellitus, and coronary artery disease) and infectious diseases (e.g., influenza, HIV, and COVID-19). Like EVs from other cell types, platelet-derived EVs (pEVs) carry diverse cargo (e.g., RNA, cytokines, and growth factors), which can be transferred to cellular receptors. Thus, because pEVs reach organs and tissues inaccessible to platelets, they may contribute to more distant cellular communication.

Studies evaluating the role of EVs in diseases caused by protozoa, such as cutaneous leishmaniasis, are scarce, and they were regarded as the function of EVs produced and released by the *Leishmania* protozoa itself [31,32,33]. In this context, the current study has used nano-flow cytometry and plasma samples from CL patients and healthy individuals to elucidate the contribution of platelet-derived MVs (pMVs) in the CL pathophysiogenesis, correlating it with the acute inflammatory phase and with antimonial treatment against the pathogen. 

In a pre-analytical approach, we tested blood collection tube protocols containing different anticoagulants (EDTA, sodium citrate, and heparin), in order to verify the one that would deliver a higher concentration of MVs in plasma samples obtained from the peripheral blood of patients with CL and healthy individuals. Sodium citrate-containing tubes showed the highest concentration of MVs when compared to heparin and EDTA tubes. This observation was corroborated by others, who stated that the use of sodium citrate prevents platelet activation in the collection process and leukocyte degranulation, consequently allowing an analysis of MVs specifically formed through the different pathophysiological states of the organism [41]. We also observed that the freezing process reduced MVs’ frequency and concentration when compared to fresh plasma. Thus, to avoid this issue we decided to perform all experiments with fresh plasma samples. 

With advances in flow cytometry, especially with regard to technology improving the nanometric sensitivity of flow cytometers, this methodology has become a fundamental tool for detecting and phenotyping MVs in almost all body fluids (e.g., plasma, urine, saliva, among others). Even so, the standardization of nano-cytofluorimetric protocols, flow cytometer settings, intra-assay quality controls, and the need to use some specific reagents are necessary [38]. The nano-flow cytometer used in the current study was the CytoFLEX (Beckman Coulter) equipped with 405 nm, 488 nm, and 638 nm lasers to detect up to twelve fluorescence, FSC, and three SSC parameters. In conventional flow cytometry, MVs and other biological nanoparticles are detected within the debris region, and most flow cytometers are not sensitive enough to effectively detect particles smaller than 300 nm in diameter. Flow cytometers sensitive to this purpose present APD sensors and a Violet SSC detector, which has an attenuation filter to reduce 95% of the scatter signal, to properly bring the sensitivity well into the nanoparticle range. A shorter wavelength, such as 405 nm (violet), results in greater orthogonal particle light scattering, as well as an increase in their resolution range. Nanometric particles such as MVs and viruses have their refractive index amplified using the violet laser, thereby improving their detection [39]. Together with flow cytometer accuracy, intra-assay quality controls are extremely essential for the improvement of data reliability. For this, following what has been recommended in the literature, we used the following intra-assay controls: an isotype control to check possible non-specific binding of the monoclonal antibodies used; unlabeled plasma samples; pure annexin V binding buffer (without EVs); and a tube with single-mAb-labeling [37,38]. Concerning the reagents required to evaluate MVs through flow cytometry, it is crucial to use annexin V binding buffer (An-bB) during the entire staining process since it has fewer crystals and aggregates and its composition contains calcium (Ca^2+^), which is essential for maintaining the link between annexin V and PS expressed on the MVs membrane. 

Several studies have reported the use of annexin V as an excellent biomarker for MVs since it specifically links to PS, which is translocated to the external membrane during microvesicle biogenesis. Nevertheless, it is known that annexin V could link to PS found on disrupted MVs and/or MVs debris, leading to a misinterpretation of false-positive MVs. Calcein AM is a membrane-permeable MV marker that has, its acetoxymethyl ester (AM) group, cleaved by esterase upon entering the MV, producing the membrane-impermeable fluorescent dye calcein. If there is a loss of integrity or an increase in membrane permeability, this esterase activity ceases and does not bind to calcein [42]. Thus, to avoid this misinterpretation between intact/true MVs and debris labeling, and to confirm the vesicular nature of the MVs, we co-labeled them with calcein AM and annexin V.

With this standardized and adapted nano-cytofluorimetric protocol, we were able to achieve our next objective of detecting, phenotyping, and quantifying MVs in the plasma of patients with CL and healthy individuals and correlated them with the pathophysiology of the disease. For this, we evaluated the frequency (%) and concentration (events/μL) of platelet-derived MVs (pMVs) in plasma samples from patients before (PBT), during (PDT), and after (PPT) treatment. We observed significantly higher levels of pMVs in the PBT group compared to the PDT groups, PPT group, and HI group, which strongly suggest that there is a direct association of these higher frequencies with the acute and inflammatory state of the disease. Conversely, when these patients were subjected to treatment with antimony, these frequencies decreased significantly, reaching almost physiological levels after the end of treatment, relating them to the beneficial effect of the treatment. 

The important role of pMVs as carriers of cytokines, lipids, proteins, nucleic acids, and organelles, which are involved in many other biological processes, to cellular recipients has already been well defined [43]. Because pMVs might reach organs and tissues inaccessible to platelets, such as joints, lymph, and bone marrow, they may contribute to the dissemination of platelet components into tissues and organs localized in sites beyond the blood system [26,27]. As they participate in the inflammatory process through their impact on cell–cell interaction, carrying pro-inflammatory cytokines, such as IL-1, IL-6, and TNF-α, which have the role of attracting macrophages and neutrophils to the inflammatory lesion site, we may speculate that these unstimulated macrophages arriving in the lesion area could act as naïve hosts being infected by *Leishmania* amastigotes, spreading and maintaining the acute pro-inflammatory status. Moreover, some studies revealed that neutrophils have been detected in patients with active cutaneous lesions of cutaneous leishmaniasis from *L. braziliensis* patients [44] and were shown to be less activated, contributing to parasite survival and dissemination [45]. The current study is in agreement with others that state that platelet-derived EVs participate in the inflammatory response during several disease manifestations such as coagulation disorders, rheumatoid arthritis, and infections by promoting leukocyte recruitment to the site of infection [43]. It is important to highlight that the levels of pMVs in plasma samples decreased when patients underwent specific treatment, reaching levels similar to the physiological state at the end of therapy. These findings suggest an association between low levels of pMVs and the resolution of the disease resulting from the antimony treatment. 

Many questions about the mechanism of action of MVs in the pathophysiology of human cutaneous leishmaniasis still remain open. However, based on the observations obtained in the current study, we may, for the first time, highlight the impact of pMVs in modulating the immune response of CL patients, relating their higher levels to the acute inflammatory state of the disease, and a significant reduction to the beneficial outcomes of the treatment. Furthermore, this knowledge is essential for defining potential treatment protocols, as well as highlighting pMVs as biomarkers for the different clinical phases of human cutaneous leishmaniasis.

## Figures and Tables

**Table 1:** Characteristics of the 40 patients with cutaneous leishmaniasis who participated in this study.

		Clinical Status	
Variable	PBT	PDT	PPT
(*n*)	18	12	10
Sex			
Female	7	5	5
Male	11	7	5
Age			
(mean ± SD) (Min–Max)	34.06 ± 14.25 (18–60)	42.83 ± 15.60 (20–60)	31.60 ± 14.40 (18–57)

PBT, patients before treatment; PDT, patients during treatment (PDT); PPT, patients at the end of treatment; HI, healthy individuals.

## Data Availability

Research data are available in tables and figures from the manuscript. Table 1 (clinical and epidemiological information) is also kept in our records if necessary.

## Supplementary Materials

The following supporting information can be downloaded at https://www.mdpi.com/article/10.3390/microorganisms12030526/s1, Figure S1: Representative nano-FCM analysis of Megamix-Plus SSC and Megamix-Plus FSC size-defined beads; (A) VSSC-logH histogram showing peaks relative to Gigamix-beads’ diameters ranging from 100 to 900 nm; (B) VSSC-logH vs. SSC-logH dot plot, where a region was created encompassing Gigamix-beads’ sizes range (100–900 nm); Figure S2: Impact of −80 °C freezing storages on the EVs’ frequency (%) and concentration (events/µL) in plasma samples. Plasma samples were analyzed fresh, after 1 day (1 d), and after at least 60 days (>60 d) at −80 °C-freezing storages. (A) Frequency (%) of MVs (annexin^+^/calcein^+^) in plasma samples; (B) concentration (events/µL) of MVs (annexin^+^/calcein^+^) in plasma samples. Dispersion graphic representing the median of 5 experiments (*n* = 5). Statistical comparisons were performed with the Mann–Whitney U-test. * *p* ≤ 0.05, ** *p* < 0.01.

## References

[1] BRASIL, Ministério da Saúde (2017). Manual de Vigilância da Leishmaniose Tegumentar. https://bvsms.saude.gov.br/bvs/publicacoes/manual_vigilancia_leishmaniose_tegumentar.pdf.

[2] OPAS, OMS Américas. https://www.paho.org/pt/topicos/leishmaniose.

[3] BRASIL, Ministério da Saúde DATASUS. http://tabnet.datasus.gov.br/cgi/tabcgi.exe?sinannet/cnv/ltarj.def.

[4] BRASIL, Ministério da Saúde Distribuição da Leishmaniose Tegumentar. https://www.gov.br/saude/pt-br/assuntos/saude-de-a-a-z/l/lt/situacao-epidemiologica.

[5] Gabriel A., Valério-Bolas A., Palma-Marques J., Mourata-Gonçalves P., Ruas P., Dias-Guerreiro T., Santos-Gomes G. (2019). Cutaneous Leishmaniasis: The Complexity of Host’s Effective Immune Response against a Polymorphic Parasitic Disease. J. Immunol. Res..

[6] Pirmez C., Yamamura M., Uyemura K., Paes-Oliveira M., Conceição-Silva F., Modlin R.L. (1993). Cytokine patterns in the pathogenesis of human leishmaniasis. J. Clin. Investig..

[7] Santiago M.A., De Luca P.M., Bertho Á.L., Azeredo-Coutinho R.B., Coutinho S.G. (2000). Detection of intracytoplasmic cytokines by flow cytometry. Mem. Inst. Oswaldo Cruz.

[8] Ruiz J.H., Becker I. (2007). CD8 cytotoxic T cells in cutaneous leishmaniasis. Parasite Immunol..

[9] Bertho A., Santiago M., Da-Cruz A., Coutinho S. (2000). Detection of early apoptosis and cell death in T CD4^+^ and CD8^+^ cells from lesions of patients with localized cutaneous leishmaniasis. Braz. J. Med. Biol. Res..

[10] Da-Cruz A.M., Oliveira-Neto M.P., Bertho Á.L., Mendes-Aguiar C.O., Coutinho S.G. (2010). T Cells Specific to *Leishmania* and Other Nonrelated Microbial Antigens Can Migrate to Human Leishmaniasis Skin Lesions. J. Investig. Dermatol..

[11] Ferraz R., Cunha C.F., Gomes-Silva A., O Schubach A., Pimentel M.I.F., Lyra M.R., Mendonça S.C., Valete-Rosalino C.M., Da-Cruz A.M., Bertho Á.L. (2015). Apoptosis and frequency of total and effector CD8+ T lymphocytes from cutaneous leishmaniasis patients during antimonial therapy. BMC Infect. Dis..

[12] Cunha C.F., Ferraz R., Pimentel M.I.F., Lyra M.R., Schubach A.O., Da-Cruz A.M., Bertho Á.L. (2016). Cytotoxic cell involvement in human cutaneous leishmaniasis: Assessments in active disease, under therapy and after clinical cure. Parasite Immunol..

[13] Cunha C.F., Ferraz-Nogueira R., Costa V.F.A., Pimentel M.I.F., Chometon T.Q., Lyra M.R., Schubach A.O., Da-Cruz A.M., Bertho A.L. (2020). Contribution of Leishmania braziliensis antigen-specific CD4^+^ T, CD8^+^ T, NK and CD3+CD56+NKT cells in the immunopathogenesis of cutaneous leishmaniasis patients: Cytotoxic, activation and exhaustion profiles. PLoS ONE.

[14] Ferraz R., Cunha C.F., Pimentel M.I.F., Lyra M.R., Pereira-Da-Silva T., Schubach A.O., Da-Cruz A.M., Bertho A.L. (2017). CD3+CD4negCD8neg (double negative) T lymphocytes and NKT cells as the main cytotoxic-related-CD107a+ cells in lesions of cutaneous leishmaniasis caused by *Leishmania* (*Viannia*) braziliensis. Parasites Vectors.

[15] Faria D.R., Souza P.E.A., Durães F.V., Carvalho E.M., Gollob K.J., Machado P.R., Dutra W.O. (2009). Recruitment of CD8+ T cells expressing granzyme A is associated with lesion progression in human cutaneous leishmaniasis. Parasite Immunol..

[16] de Oliveira C.I., Brodskyn C.I. (2012). The immunobiology of *Leishmania braziliensis* infection. Front. Immunol..

[17] Brahim L.R., Valete-Rosalino C.M., Antônio L.d.F., Pimentel M.I.F., Lyra M.R., Paes L.E.d.C., da Costa A.D., Vieira I.F., Dias C.M.G., Duque M.C.d.O. (2017). Low dose systemic or intralesional meglumine antimoniate treatment for American tegumentary leishmaniasis results in low lethality, low incidence of relapse, and low late mucosal involvement in a referral centre in Rio de Janeiro, Brazil (2001–2013). Mem. Inst. Oswaldo Cruz.

[18] Cataldo J.I., Conceição-Silva F., Antônio L.d.F., Schubach A.d.O., Marzochi M.C.d.A., Valete-Rosalino C.M., Pimentel M.I.F., Lyra M.R., Oliveira R.d.V.C.d., Barros J.H.d.S. (2018). Favorable responses to treatment with 5 mg Sbv/kg/day meglumine antimoniate in patients with American tegumentary leishmaniasis acquired in different Brazilian regions. Rev. Soc. Bras. Med. Trop..

[19] Saheki M.N., Lyra M.R., Bedoya-Pacheco S.J., Antônio L.d.F., Pimentel M.I.F., Salgueiro M.d.M., Vasconcellos É.d.C.F.e., Passos S.R.L., dos Santos G.P.L., Ribeiro M.N. (2017). Low versus high dose of antimony for American cutaneous leishmaniasis: A randomized controlled blind non-inferiority trial in Rio de Janeiro, Brazil. PLoS ONE.

[20] Wang S., Yang J., Zhao G., Liu R., Du Y., Cai Z., Luan J., Shen Y., Chen B. (2021). Current applications of platelet gels in wound healing—A review. Wound Repair Regen..

[21] Shadmand E., Solhjoo K., Taghipour A., Tayer A.H., Sadeghi F., Meshkin A. (2023). Healing effects of autologous platelet gel and growth factors on cutaneous leishmaniasis wounds in addition to antimony; a self-controlled clinical trial with randomized lesion assignment. BMC Res. Notes.

[22] Sonmez O., Sonmez M. (2017). Role of platelets in immune system and inflammation. Porto Biomed. J..

[23] Yuana Y., Sturk A., Nieuwland R. (2013). Extracellular vesicles in physiological and pathological conditions. Blood Rev..

[24] Yáñez-Mó M., Siljander P.R.-M., Andreu Z., Bedina Zavec A., Borràs F.E., Buzas E.I., Buzas K., Casal E., Cappello F., Carvalho J. (2015). Biological properties of extracellular vesicles and their physiological functions. J. Extracell. Vesicles.

[25] Heijnen H.F., E Schiel A., Fijnheer R., Geuze H.J., Sixma J.J. (1999). Activated platelets release two types of membrane vesicles: Microvesicles by surface shedding and exosomes derived from exocytosis of multivesicular bodies and alpha-granules. Blood.

[26] Puhm F., Boilard E., Machlus K.R. (2021). Platelet Extracellular Vesicles. Arter. Thromb. Vasc. Biol..

[27] Mabrouk M., Guessous F., Naya A., Merhi Y., Zaid Y. (2022). The Pathophysiological Role of Platelet-Derived Extracellular Vesicles. Semin. Thromb. Hemost..

[28] Puhm F., Flamand L., Boilard E. (2022). Platelet extracellular vesicles in COVID-19: Potential markers and makers. J. Leukoc. Biol..

[29] French S.L., Butov K.R., Allaeys I., Canas J., Morad G., Davenport P., Laroche A., Trubina N.M., Italiano J.E., Moses M.A. (2020). Platelet-derived extracellular vesicles infiltrate and modify the bone marrow during inflammation. Blood Adv..

[30] Hua Q., Lyonb C.J., Fletcher J.K., Tanga W., Wana M., Hub T.Y. (2021). Extracellular vesicle activities regulating macrophage- and tissue-mediated injury and repair responses. Acta Pharm. Sin. B.

[31] Silverman J.M., Clos J., Horakova E., Wang A.Y., Wiesgigl M., Kelly I., Lynn M.A., McMaster W.R., Foster L.J., Levings M.K. (2010). Leishmania Exosomes Modulate Innate and Adaptive Immune Responses through Effects on Monocytes and Dendritic Cells. J. Immunol..

[32] Silverman J.M., Reiner N.E. (2012). Leishmania Exosomes Deliver Preemptive Strikes to Create an Environment Permissive for Early Infection. Front. Cell. Infect. Microbiol..

[33] Emerson L.E., Gioseffi A., Barker H., Sheppe A., Morrill J.K., Edelmann M.J., Kima P.E. (2022). Leishmania infection-derived extracellular vesicles drive transcription of genes involved in M2 polarization. Front. Cell. Infect. Microbiol..

[34] Leite-Silva J., Oliveira-Ribeiro C., Morgado F.N., Pimentel M.I.F., Lyra M.R., Fagundes A., Miranda L.F.C., Valete-Rosalino C.M., Schubach A.O., Conceição-Silva F. (2023). Is There Any Difference in the In Situ Immune Response in Active Localized Cutaneous Leishmaniasis That Respond Well or Poorly to Meglumine Antimoniate Treatment or Spontaneously Heal?. Microorganisms.

[35] Filgueira C.P.B., Moreira O.C., Cantanhêde L.M., de Farias H.M.T., Porrozzi R., Britto C., Boité M.C., Cupolillo E. (2020). Comparison and clinical validation of qPCR assays targeting *Leishmania* 18S rDNA and HSP70 genes in patients with American Tegumentary Leishmaniasis. PLoS Neglected Trop. Dis..

[36] Wisgrill L., Lamm C., Hartmann J., Preißing F., Dragosits K., Bee A., Hell L., Thaler J., Ay C., Pabinger I. (2016). Peripheral blood microvesicles secretion is influenced by storage time, temperature, and anticoagulants. Cytom. Part A.

[37] Théry C., Witwer K.W., Aikawa E., Alcaraz M.J., Anderson J.D., Andriantsitohaina R., Antoniou A., Arab T., Archer F., Atkin-Smith G.K. (2018). Minimal information for studies of extracellular vesicles 2018 (MISEV2018): A position statement of the International Society for Extracellular Vesicles and update of the MISEV2014 guidelines. J. Extracell. Vesicles.

[38] Welsh J.A., Arkesteijn G.J.A., Bremer M., Cimorelli M., Dignat-George F., Giebel B., Görgens A., Hendrix A., Kuiper M., Lacroix R. (2023). A compendium of single extracellular vesicle flow cytometry. J. Extracell. Vesicles.

[39] Brittain G.C., Chen Y.Q., Martinez E., Tang V.A., Renner T.M., Langlois M.-A., Gulnik S. (2019). A Novel Semiconductor-Based Flow Cytometer with Enhanced Light-Scatter Sensitivity for the Analysis of Biological Nanoparticles. Sci. Rep..

[40] Carnino J.M., Lee H., Jin Y. (2019). Isolation and characterization of extracellular vesicles from Broncho-alveolar lavage fluid: A review and comparison of different methods. Respir. Res..

[41] Mullier F., Bailly N., Chatelain C., Chatelain B., Dogné J. (2013). Pre-analytical issues in the measurement of circulating microparticles: Current recommendations and pending questions. J. Thromb. Haemost..

[42] Gray W.D., Mitchell A.J., Searles C.D. (2015). An accurate, precise method for general labeling of extracellular vesicles. MethodsX.

[43] Melki I., Tessandier N., Zufferey A., Boilard E. (2017). Platelet microvesicles in health and disease. Platelets.

[44] Conceição J., Davis R., Carneiro P.P., Giudice A., Muniz A.C., Wilson M.E., Carvalho E.M., Bacellar O. (2016). Characterization of Neutrophil Function in Human Cutaneous Leishmaniasis Caused by Leishmania braziliensis. PLoS Neglected Trop. Dis..

[45] Cardoso T., Bezerra C., Medina L.S., Ramasawmy R., Scheriefer A., Bacellar O., de Carvalho E.M. (2019). Leishmania braziliensis isolated from disseminated leishmaniasis patients downmodulate neutrophil function. Parasite Immunol..

