# Supplementary figures and images for: Platelet-Derived Microvesicles Contribute to the Pathophysiogenesis of Human Cutaneous Leishmaniasis: A Nano-Flow Cytometric Approach in Plasma Samples from Patients before and under Antimonial Treatment
Source: Microorganisms. 2024 Mar 6;12(3):526. doi: 10.3390/microorganisms12030526 (PMC10975300; doi:10.3390/microorganisms12030526)

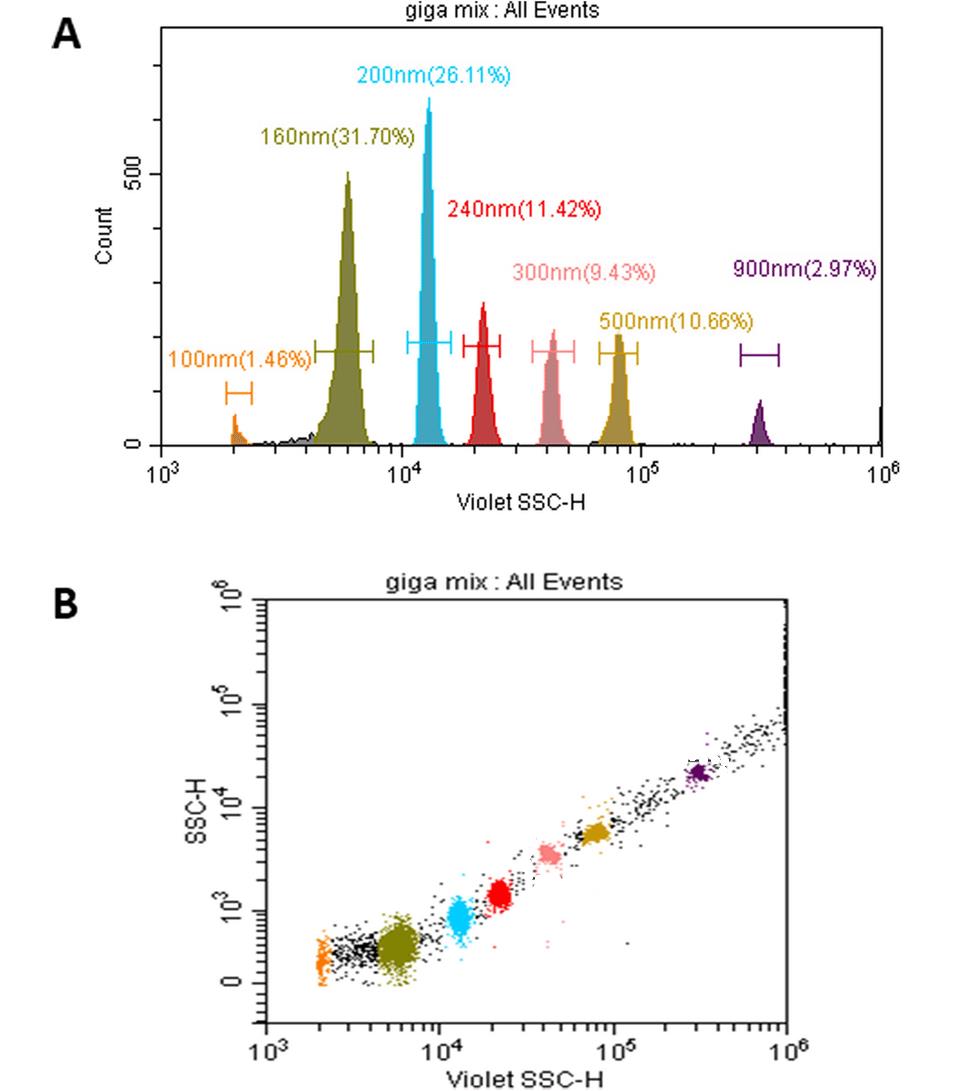

Supplement: Supplementary file 1 [file microorganisms-12-00526-s001.zip › Suppl Figure S1.jpg]

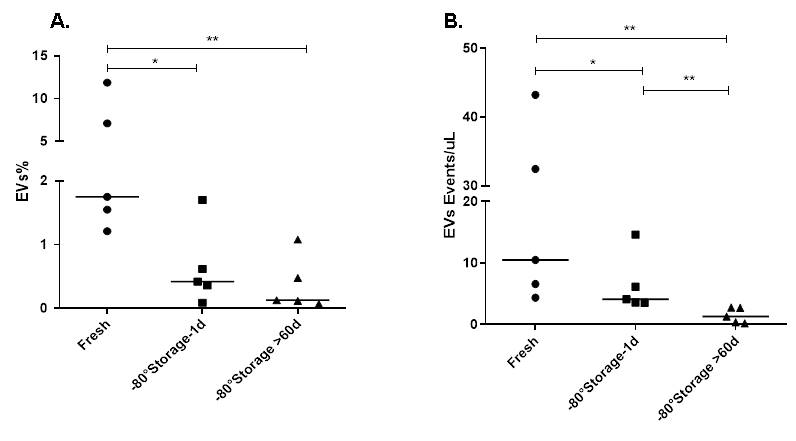

Supplement: Supplementary file 1 [file microorganisms-12-00526-s001.zip › Suppl Figure S2.jpg]
